# The Effect of Vitamin D Deficiency on Post-operative Outcomes Following Rotator Cuff Repair: A Meta-Analysis

**DOI:** 10.7759/cureus.90644

**Published:** 2025-08-21

**Authors:** Alwaleed A Alshahir, Khalid Alorf, Faisal Alhabradi, Faisal H AlNaqa, Abdulmalik Alanazy, Khalid A Mani, Abdulaziz Almadani, Ahmed Alhussain

**Affiliations:** 1 Orthopedic Surgery, King Abdullah International Medical Research Centre, Riyadh, SAU; 2 Orthopedics, King Abdullah International Medical Research Centre, Riyadh, SAU; 3 Orthopedic Surgery, King Saud bin Abdulaziz University for Health Sciences, Riyadh, SAU; 4 Orthopedics, King Abdulaziz Medical City, Riyadh, SAU; 5 Orthopedics, Prince Sultan Military Medical City, Riyadh, SAU; 6 Orthopedic Surgery, King Abdulaziz Medical City Riyadh, Riyadh, SAU; 7 Orthopedics, King Saud bin Abdulaziz University for Health Sciences, Riyadh, SAU; 8 Orthopedics, Ministry of National Guard Health Affairs (MNGHA), Riyadh, SAU

**Keywords:** arthroscopy, outcome, rotator cuff, shoulder, vitamin d

## Abstract

Rotator cuff injuries (RCIs) are common and debilitating injuries, particularly among older adults. Surgical repair, such as arthroscopic rotator cuff repair (RCR), is the preferred treatment, with pre-operative risk factors, including vitamin D deficiency, associated with poor outcomes, but the relationship between vitamin D deficiency and outcomes following RCR remains uncertain. The purpose of this meta-analysis was to investigate the relation between pre-operative vitamin D deficiency and post-operative outcomes following RCR. A systematic review and meta-analysis were conducted to explore the association including randomized clinical trials, observational studies, and case-series with more than 10 adult participants (age > 18) evaluating post-operative outcomes in vitamin D-deficient RCR patients. Six studies encompassing 44,546 patients with RCIs were included. Vitamin D deficiency was significantly associated with an increased risk of surgery revision (RR 1.220; 95% CI 1.133, 5.245; *p* < .001). However, there was no significant correlation between vitamin D deficiency and post-operative pain (*p* = .064), tear size (*p* = .480), or functional scores, including ASES (*p* = .820), Constant score (*p* = .365), and UCLA score (*p* = .780). This study revealed that vitamin D deficiency increased the risk of surgery revision to address repair failure following RCR by 22% in vitamin D-deficient patients. No significant associations were found with post-operative pain, which could be related to the limited number of studies. Further studies are still needed to yield more conclusive results.

## Introduction and background

Introduction

Rotator cuff injuries (RCIs) are one of the most common disabling injuries in the upper limb. The role of the aging process plays a major risk factor in its injury [1,2]. As reported in recent studies, approximately 30% of adults over 60 years of age have rotator cuff tears, and around 62% of adults over the age of 80 have tears (whether or not symptoms are present) [2]. Generally, patients with RCIs present with clinical manifestations of shoulder pain and/or dysfunction that compromise their quality of life [3]. Although surgical treatment for rotator tears with arthroscopic rotator cuff repair (RCR) remains the treatment of choice as it helps in relieving the pressure on the rotator cuff tendon and improving symptoms, variable patient-reported outcomes are considered important to assess [4-6]. For instance, pain and retear following such an operation are of primary concern to patients [4-7].

In the literature, several studies found a number of pre-operative risk factors that have a role in the outcome of RCR failure such as age, tear size, diabetes, osteoporosis, and/or vitamin D deficiency [8,9]. Due to the high prevalence of vitamin D deficiency among individuals undergoing arthroscopic rotator cuff surgery, recent research has increasingly focused on its potential role in patient outcomes [10]. The effects of vitamin D levels have been reported to be not only the regulation of bone mineralization and fracture healing, but also seem to be involved in articular cartilage function and soft tissue healing and strength [11]. Vitamin D contributes to bone and soft tissue healing by modulating inflammatory responses, enhancing antimicrobial activity, and stimulating fibroblast-mediated collagen synthesis. It supports angiogenesis, facilitates muscle regeneration, and regulates matrix remodeling to limit excessive scar formation. Deficiency is associated with delayed healing and impaired bone and soft tissue quality [11]. Previously, a study found that vitamin D deficiency is associated with post-operative complications after total knee arthroplasty [12].

Therefore, it is important to evaluate the association between existing osteoporosis and vitamin D levels and the common adverse outcomes after RCR. Since there are undefined common results in the literature regarding whether low vitamin D levels have a role related to the complications after RCR, the authors believed that a meta-analysis of the existing studies in the literature is needed, which has not been published previously in the literature. Therefore, the purpose of this meta-analysis is to investigate the relation between pre-operative vitamin D deficiency and post-operative complications following RCR.

## Review

Materials and methods

Literature Review

This systematic review and meta-analysis was conducted in line with the Preferred Reporting Items for Systematic Reviews and Meta-Analysis (PRISMA) guidelines [13,14]. In July 2023, we undertook a comprehensive review of literature and a meta-analysis, aiming to illustrate the effect of vitamin D deficiency on post-operative complications following RCR.

Several databases were used to conduct this review, including Web of Science, Medline, EBSCO, ProQuest, Embase, and Scopus without any date restriction. The literature was searched using the following search strategy: (Vitamin D deficiency OR Vitamin D low level OR Vitamin D) AND (Rotator cuff OR Rotator Cuffs OR Teres Minor OR Subscapular is OR Infraspinatus OR Supraspinatus OR rotator cuff arthroscopic repair) AND (Rotator Cuff Injury OR Rotator Cuff Injuries OR Rotator Cuff Tears OR Rotator Cuff Tear OR Postoperative Complication OR Postoperative Complications OR Cuff Tear Arthropathy OR shoulder stiffness OR pain OR revision surgery OR revision OR complication OR complications OR retear OR outcomes OR outcome).

The electronic databases were screened from their inception to July 2023. The review was registered in the International Prospective Register of Systematic Reviews (Registration ID: CRD42023439220) for transparency and traceability [13]. This study was carried out in alignment with the Declaration of Helsinki, and owing to the research methodological nature, no ethical approval was required.

Study Selection

The study inclusion criteria were limited to studies with the aim of evaluating post-operative complications following RCR in vitamin d deficient adult patients (age > 18 years), studies published in English peer review journals with a study design of randomized clinical trials (RCTs), observational studies, and case-series studies with a sample size of more than 10 patients. We excluded systematic review or meta-analysis studies, studies published in a language other than English, improper methods, no outcome of interest, animal studies, cadaveric studies, and editorial papers. We used the Rayyan search engine to screen all titles and abstracts of the extracted studies, adhering to our set inclusion and exclusion criteria. All extracted studies were divided into two groups, with each group being screened by two independent reviewers. Any disagreements between the articles were resolved by reaching a consensus with a fifth independent reviewer. Subsequently, both groups assessed full-text articles for compliance with the inclusion and exclusion criteria.

Data Extraction and Measurement

The extracted parameters from the articles were: title, author’s last name, year of publication, name of journal, country, study design, definition of vitamin d deficiency, sample size, age, sex, body mass index, diabetes, smoking, hypertension, peripheral vascular disease, shoulder functional scores like the constant score, and the University of California-Los Angeles score (UCLA), post-operative pain, tear size, and surgery revision rate. The primary outcome of interest in this review was post-operative revision surgery as complications following RCR.

Risk of Bias

Two researchers in the study used the Newcastle-Ottawa Scale, a widely recognized tool for evaluating the methodological rigor of the selected studies [15]. Based on this scale, each study is judged according to eight items, which are divided into three components. The selection of study groups, the compatibility of the groups, and the ascertainment of exposure or outcome of interest. A star system recorded helps by judging the quality level of each study with the highest quality studies show an adequate selection of study groups with a maximum score of 4 stars, an established comparability on the basis of the design or analysis for either cases and controls with a maximum score of two stars, and an assessment of the outcome or exposure adequacy with a maximum score of three stars [16,17]. For more information, Table 1 summarizes the risks of bias assessment for all the included studies.

**Table 1:** Newcastle-Ottawa Scale (NOS) assessment tool for risk of bias in non-randomized studies in meta-analysis

Study	Selection				Comparability	Outcome			Quality score
	Representativeness Of the exposed cohort	Selection of the non-exposed cohort	Ascertainment of exposure	Demonstration that outcome of interest was not present at start of study	Comparability of cohorts on the basis of the design or analysis	Assessment of outcome	Was follow-up long enough for outcomes to occur	Adequacy of follow up of cohorts	None
Cancienne et al. [18]	*	*	*	*	**	*	*	*	good quality
Chen et al. [19]	*	*	*	*	**	*	*	*	good quality
Harada et al. [20]	*	*	*	*	None	*	*	*	Poor quality
O’Donnell et al. [21]	*	None	*	None	**	*	*	*	fair quality
Rhee et al. [22]	*	None	*	*	None	*	*	*	fair quality
Ryu et al. [23]	*	*	*	*	**	*	*	*	good quality

Statistical Analysis

Risk ratio (RR) and the 95% confidence interval (95% CI) was employed to express dichotomous outcomes. For the assessment of the association between vitamin D deficiency and surgical as well as functional outcomes, a pooled correlation coefficient (r) was derived from a meta-analysis of effect sizes from all included studies.

In the case where the population effect size is assumed constant, the fixed-effect model was applied. On the other hand, a random-effects model was employed in cases where statistical heterogeneity existed. Heterogeneity was decided using Higgins' I² statistic and the Cochrane Q (Chi² test), and thresholds were >50% for I² and p < .10 for the Chi² test [16,17]. All data analyses were done using Comprehensive Meta-Analysis version 3 software, and statistical significance was considered at p < .05.

Results

General Characteristics of the Included Studies

**Figure 1:**
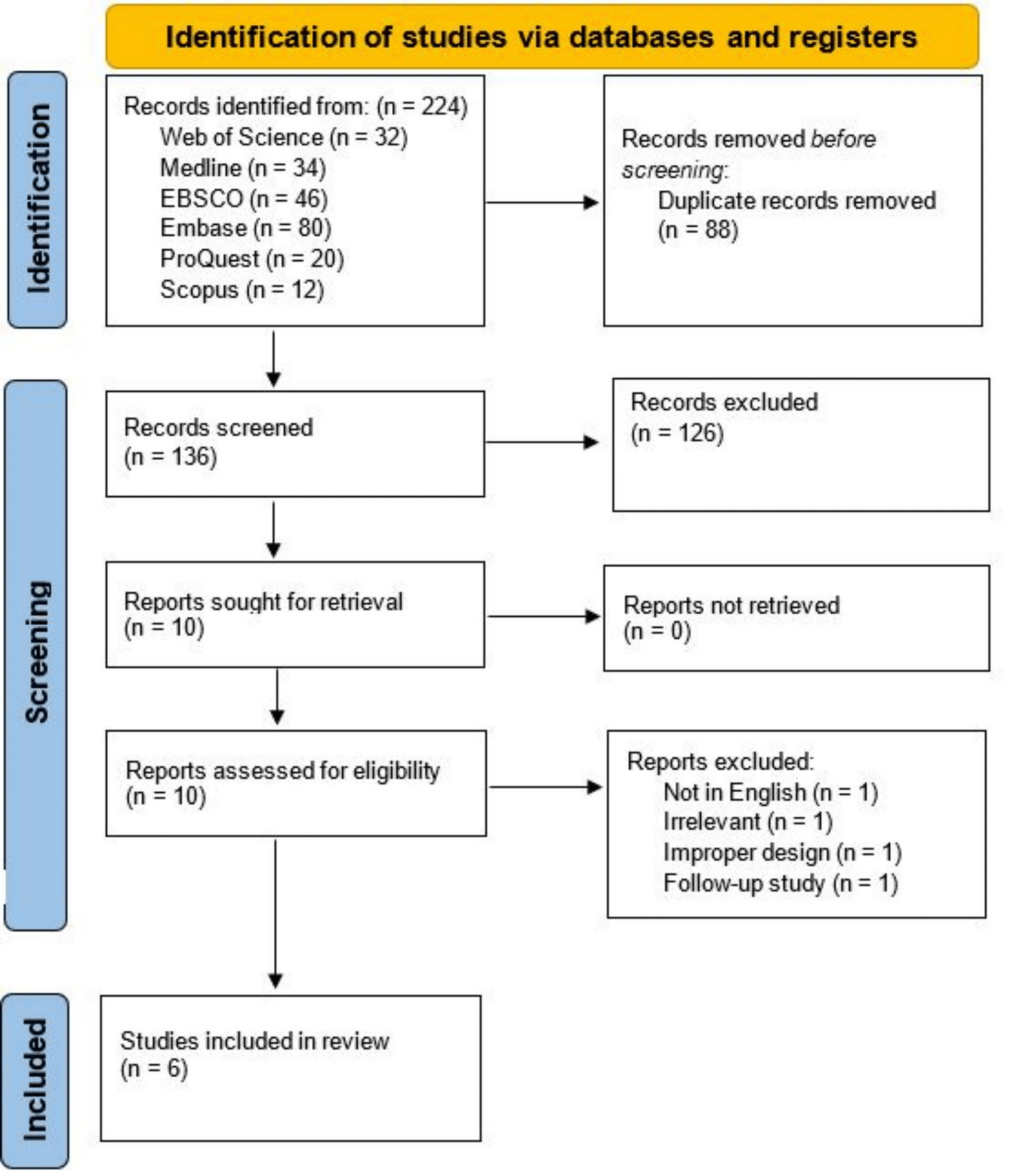
The Preferred Reporting Items for Systematic Reviews and Meta-Analyses (PRISMA) flowchart of the selection process.

Following our comprehensive search of the six databases, 224 studies were initially found. After removing 88 duplicates, we screened the titles and abstracts and found 10 studies eligible for full-text screening. No studies were found on the manual search of reference lists. Additionally, four studies were excluded from full-text screening due to irrelevance (n = 1), improper design (n = 1), full-text not in English (n = 1), and follow-up study with no significant data to be included (n = 1). Therefore, only six studies were included in the qualitative synthesis based on our inclusion and exclusion criteria [18-23]. The detailed Preferred Reporting Items for Systematic Reviews and Meta-Analyses (PRISMA) flowchart of the selection process is presented in Figure 1.

Demographic Characteristics of the Included Studies

The current meta-analysis incorporated six studies [18-23], involving a total of 44,546 patients diagnosed with rotator cuff tear injuries. Three studies included patients from the USA, while two articles included patients from the Republic of Korea. Four articles were retrospective design, and two were prospective cohort design. There were 33900 patients with normal vitamin D levels, while 10646 patients had abnormal levels. The average age of the included patients ranged from 60.61 to 61.78 years. There were 1434 and 468 females among patients with normal and abnormal vitamin D levels, respectively. There were 817 obese patients and 152 smokers among the included patients. Furthermore, there were 860 diabetic patients among the normal vitamin D group, in contrast to 320 patients among the abnormal vitamin D group. There were 602 patients with peripheral vascular diseases as shown in Table 2.

**Table 2:** Demographic characteristics of the included studies PVD: Peripheral vascular disease; NR: Non-reported; SD: Standard deviation

Study ID		Study Region	Study Design	Study Period	Sample Size		Age (Years)		Gender				Comorbidities								
									Female		Male		Obesity		Smoking			Diabetes Mellitus		PVD	
					Normal	Abnormal	Normal	Abnormal	Normal	Abnormal	Normal	Abnormal	Normal	Abnormal	Normal	Abnormal	Normal		Abnormal	Normal	Abnormal
					Number	Number	Mean ±SD	Mean ±SD	Number	Number	Number	Number	Number	Number	Number	Number	Number		Number	Number	Number
1	Cancienne et al., 2019 [18]	USA	Retrospective	NR	541	441	NR	NR	355	287	186	154	121	140	65	68	226		198	76	69
2	Chen et al., 2022 [19]	China	Retrospective	January 2018 and August 2019	44	45	60.61 ± 7.62	61.78 ± 7.29	25	28	19	17	NR	NR	10	9	4		5	NR	NR
3	Harada et al., 2019 [20]	USA	Retrospective	2007 and 2016	1,652	229	NR	NR	1,054	153	598	76	473	86	NR	NR	630		117	395	62
4	O’Donnell et al., 2020 [21]	USA	Retrospective	January 1, 2007, to April 1, 2016	31657	9,810	NR	NR	NR	NR	NR	NR	NR	NR	NR	NR	NR		NR	NR	NR
5	Rhee et al., 2023 [22]	Republic of Korea	Prospective Cohort	March 2017 to October 2017	3	33	NR	NR	NR	NR	NR	NR	NR	NR	NR	NR	NR		NR	NR	NR
6	Ryu et al., 2015 [23]	Republic of Korea	Prospective Cohort	December 2011 to June 2013	3	88	NR	NR	NR	NR	NR	NR	NR	NR	NR	NR	NR		NR	NR	NR

Vitamin D Measurement and Cut-off

Among the included studies, two studies categorized patients into deficient and normal vitamin D (deficient= <20ng/mL, normal = >20ng/mL) [19,20]. Two studies categorized patients into deficient, insufficient, and normal vitamin D (deficient = <20ng/mL, insufficient = 20-30ng/mL, normal = >30ng/mL) [18-23]. One study followed the same categorization of deficient, insufficient, and normal vitamin D, however, with different cut-off (deficient = <20ng/mL, Insufficient = 10-20ng/mL, normal>20ng/mL) while the last study did not report any information on vitamin D levels [21]. In this review, we classified patients in terms of vitamin D levels into normal and abnormal (deficient and insufficient).

Revision Surgery

**Figure 2:**
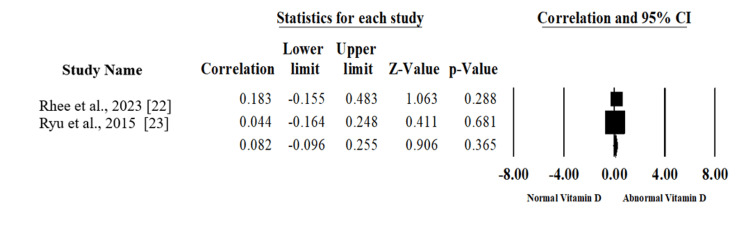
Forest plot of summary analysis of the risk ratio and 95% confidence interval (CI) of the risk of revision surgery after rotator cuff repair between patients with normal and abnormal levels of vitamin D. Size of the black squares is proportional to the statistical weight of each trial. The grey diamond represents the pooled point estimate. The positioning of both diamonds and squares (along with 95% CIs) beyond the vertical line (unit value) suggests a significant outcome (IV = inverse variance).

Four articles included 44419 patients with RCR, which evaluated the risk of revision surgery [18-22]. There were 33894 patients with normal vitamin D levels, while 10525 patients had abnormal levels. When the data were pooled using the random-effects model (I² = 0%, p = .398), the results indicated a statistically significant increased risk of repair revision surgery among patients with vitamin D deficiency with a risk ratio of 1.220 (95%CI; 1.133, 5.245; p < .001), as demonstrated in Figure 2, which was created using data from [22,23]. 

Vitamin D Deficiency and Pain

**Figure 3:**
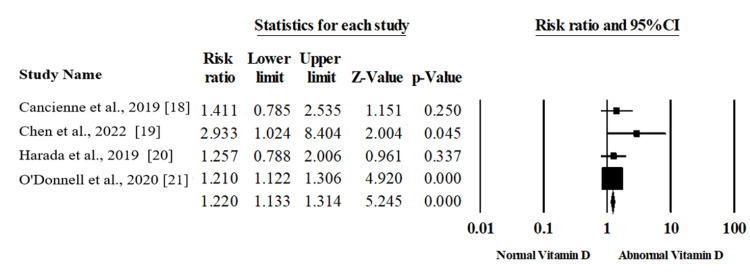
Forest plot of summary analysis of the correlation and 95% confidence interval (CI) of the association between levels of vitamin D and post-operative pain. The size of the black squares is proportional to the statistical weight of each trial. The grey diamond represents the pooled point estimate. The positioning of both diamonds and squares (along with 95% CIs) beyond the vertical line (unit value) suggests a significant outcome (IV = inverse variance).

 Four studies included 125 patients evaluating the correlation between vitamin D levels and the severity of post-operative pain [18-21]. There was no statistically significant (p=.064) association between the levels of vitamin D and post-operative pain (Correlation -0.168, 95%CI; -0.336, 0.010) without heterogeneity between the included studies (I2=0 %, p=.576) as shown in Figure 3, which was created using data from [18-21].

Vitamin D Deficiency and ASES

**Figure 4:**
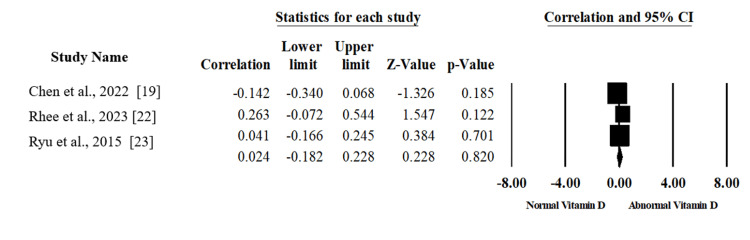
Forest plot of summary analysis of the correlation and 95% CI of the association between levels in D and American Shoulder and Elbow Surgeons score (ASES). The size of the black squares is proportional to the statistical weight of each trial. The grey diamond represents the pooled point estimate. The positioning of both diamonds and squares (along with 95% CIs) beyond the vertical line (unit value) suggests a significant outcome (IV = inverse variance).

The association between vitamin D levels and ASES score was evaluated within three articles, including 216 patients [19,22,23]. In the random-effects model (I2=53.47%, p =.117), there was no statistically significant association between the levels of vitamin D and ASES score among patients with RCIs (correlation 0.024, 95%CI; -0.182, 0.228; p=.820), as shown in Figure 4, which was created using data from [19,22,23].

Vitamin D Deficiency and Constant Score

**Figure 5:**
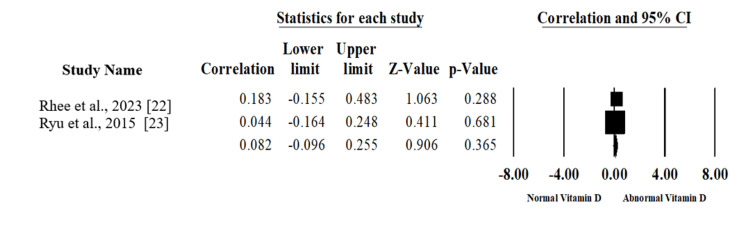
A forest plot summarizing the correlation and corresponding 95% confidence interval (CI) between vitamin D levels and the Constant score.

Two articles that included 127 patients reported the association between vitamin D levels and constant score [22,23]. There was no statistically significant association between the levels of vitamin D and constant score (Correlation 0.082, 95%CI; -0.096, 0.225; p=.365) with homogeneity between the included studies (I2=0%, p=.489) as shown in Figure 5, which was created using data from [22,23].

Vitamin D Deficiency and Tear Size

**Figure 6:**
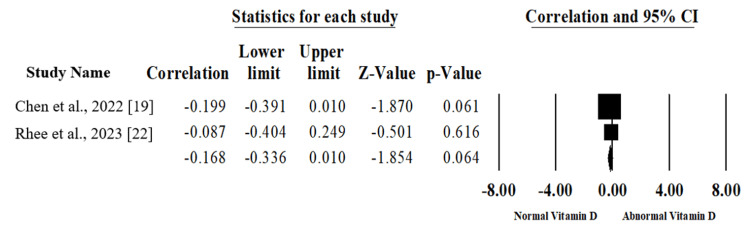
Forest plot of summary analysis of the correlation and 95% confidence interval (CI) of the association between levels of vitamin D and tear size.

Two studies evaluated the association between vitamin D deficiency and tear size among 125 patients [19,22]. There was no statistically significant correlation (p=0.480) between the levels of vitamin D and tear size with a correlation of -0.065 (95%CI; -0.240, 0.144). In the random-effects model (I2=0%, p=.626) as presented in Figure 6, which was created using data from [19,22].

Vitamin D Deficiency and UCLA Score

**Figure 7:**
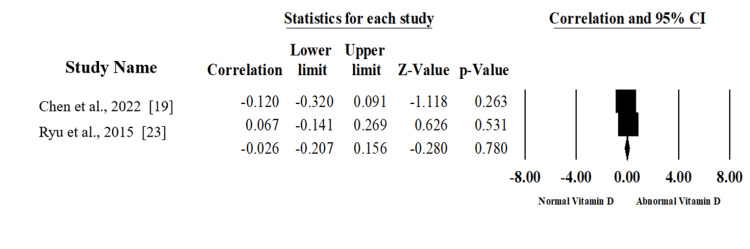
Forest plot displaying the summary analysis of the correlation (with 95% confidence intervals) between vitamin D levels and University of California-Los Angeles (UCLA) scores.

The association between vitamin D levels and University of California-Los Angeles score (UCLA) scores was evaluated within two studies, including 180 patients [19,23]. Pooling the effect sizes in the random-effects model (I2=34.5%, P=0.217) revealed no statistically significant association (correlation -0.026, 95%CI; -0.207, -0.280; p=.780) as shown in Figure 7, which was created using data from [19,23].

Discussion

The current meta-analysis included six articles to investigate the effect of vitamin D deficiency on post-operative complications following RCR procedures. Our analysis of 44546 patients who underwent RCR revealed that among several complications, surgery revision was a well-documented complication among vitamin D-deficient patients. This finding aligns with the known role and importance of vitamin D in the healing processes and tissue repair, along with the theory of the effectiveness of vitamin supplementation as a preventive measure [24-34].

Vitamin D Effects on Tissue Repair and the Healing Process

The literature has been bombarded with evidence regarding the effect of vitamin D on tissue healing and repair [31,32]. Although surgical techniques remain the most important determinant to obtain successful rotator cuff stabilization, tendon-to-bone healing failure is still a major problem [35]. Therefore, deep molecular and biological understanding of orthopedic diseases has been recently reviewed to examine the ability of the body responses to the healing process [36-38]. In controlled laboratory research, a study compared vitamin D-deficient rats with vitamin D normal control rats [39]. They analyzed the effects on the healing process of the vitamin D deficiency group after they performed a supraspinatus right tendon rupture repair surgically. The results showed a significantly low load to failure in the deficient group, in addition to having more normal tendon insertion strength in the vitamin D normal group. Furthermore, the histological assessment showed fewer collagen fibers and bone formation in the low vitamin D group. The study found that low vitamin D levels could negatively impact recovery following rotator cuff surgery [39]. In addition, several other studies demonstrated that vitamin D deficiency has inhibited the activity of acute tendon healing, and a normal vitamin D level affects skeletal muscle strength and increases bone mineral density by that having a significant role in the tendon-to-bone healing process [25,26].

Cost of Surgery Revision and Effectiveness of Supplementation

This study showed that vitamin D-deficient patients have an increased rate of revision following RCR. In addition, retrospective studies in RCR have observed an increased rate of revision in vitamin D-deficient patients compared to pre-operative vitamin D-sufficient patients [22,23]. One of the most expensive complications that has been found after RCR is the development of early retears, of which the true effect on postoperative functional outcomes remains variable [40]. As previously thought that patients with rotator cuff retear after primary RCR have little or no clinical changes, according to multiple studies including a meta-analysis. demonstrated that there are significantly lower functional scores and decreased abduction strength in patients with full-thickness tears in comparison to intact or partially torn [38-43]. Moreover, several established risk factors such as older age, diabetes, reduced bone mineral density, and fatty infiltration of the subscapularis or infraspinatus have been strongly correlated with the occurrence of retears [44,45]. However, published studies investigating the role of low vitamin D as a risk factor for revision surgery after RCR are not well defined in the literature. Four studies have concluded that vitamin D deficiency increased the rates of retear and revision surgery. First, a level 3 retrospective study published found increased rates of revision RCR in 25(OH) D-deficient patients (8.3%) compared to sufficient patients (6.6%) ([OR] 1.54; 95% [CI] 1.21-1.97; p<.001) [20]. Furthermore, a retrospective case series by O’Donnell et al. showed increased odds of revision RCR in 25(OH) D-deficient patients (8.54%) compared to 25(OH) D-sufficient patients (7.06%) ([OR] 1.18; 95% [CI] 1.08- 1.28; p< .001) [21]. In addition, a revision rate of 5.88% after RCR was also observed in the deficient group compared to a 3.7% revision rate in the sufficient group (odds ratio [OR] 3.1;95% confidence interval [CI] 1.6-5.8; p=.007 [18]. Last, in a level 3 cohort study it was shown that after surgery, the deficient group compared to the control group had a higher retear rate (26.67% vs 9.09% P<.05), and higher early pain (1.47 [0.66] vs 1.09 [0.56], respectively, at one month; 1.44 [0.66] vs 1.14 [0.77] at three months; all p<.05) [19]. Therefore, a model of vitamin D supplementation may be a cheap method to reduce the significant expense of RCR revision rates. In fact, a cost-effectiveness study proved the role of selective and non-selective vitamin D supplementation strategy in decreasing revision rates of RCR and reducing healthcare cost and overall burden of healthcare [45]. They estimated that pre-operative non-selective and selective vitamin D replacement in RCR patients is an effective cost solution and would cost a mean saving of $6,099,341 and $11,584,742 for every 2500000 cases of RCR, respectively. The study found selective supplementation to be cost-effective for clinical environments when the cost of revision RCR exceeds $14,824.69 and there is a rate of vitamin D deficiency of greater than 6.67%, and non-selective supplementation would be cost-effective for the clinical environment where there is a revision RCR cost exceeding $4,216.06 and vitamin D deficiency with a rate exceeding 1.93%. Moreover, a meta-analysis of randomized control trials published in China concluded that Vitamin D supplementation has been suggested to be effective in patients with knee osteoarthritis by both significantly reducing pain and functional score of knee osteoarthritis (OA) using the Western Ontario and McMaster Universities Arthritis Index (WOMAC). This beneficial effect has been observed in studies that recommended a daily dose of 2000 IU or more. However, they reported no reduction in the progression of knee OA and cartilage loss [46].

Other Complications

Even though this study addressed complications other than surgery revision such as post-operative pain, tear size, they were not significantly correlated with vitamin D deficiency. However, this might have been impacted by the heterogeneity of studies addressing these complications and the inconsistency of reporting them. Yet, several studies have found that pain and functional scores might be affected by vitamin D deficiency. In vitro studies have shown that vitamin D inhibits the synthesis of inflammatory mediators such as prostaglandin E2 (PGE2), which is an important factor in pain sensation [26,27]. Furthermore, previous studies have suggested a potential role of vitamin D deficiency in adverse surgical outcomes, and that vitamin D deficiency acts as a risk for these post-operative complications. A study demonstrated in their population cohort the effect of vitamin D and the revision rate after primary total knee replacement surgery [47]. The study included all patients who underwent primary total knee replacement between 2009 and 2018. The research has reviewed a total of 142,147 patients in the analysis, but only 20% of the total respondents (n= 28,403) took vitamin D and calcium-containing preparations. The results of the study confirmed that implant survival was significantly higher in patients taking a combination of vitamin D and calcium preparation (at a dose of 800 IU or higher) for more than one year in comparison to patients who did not have it. In addition, a study published in the Republic of Korea examined the post-operative function after total knee arthroplasty for patients with low serum vitamin D [4]. The study concluded that early post-operative functional outcomes and performance tests appeared to be negatively affected by vitamin D deficiency, which further connects vitamin D deficiency to poor surgical outcomes.

Limitations and Recommendations

This study faced several limitations, the first was the inconsistency of data reporting in the included studies. Several variables were not addressed and unified among studies, which limited our inclusion of more reported complications. For example, the variable of shoulder functional scores was not periodically reported for the three-month, six-month, and one-year follow-up. In addition, relater rate which was an interesting outcome to be looking at, was not reported among several studies, thus, allowing the pooled data to lead to heterogeneity and unreliable conclusion. Another limitation is the lack of high-quality level I evidence which would have aided the conclusion significantly. However, this study’s limitation can be an eye opener for future research to address our same research question by providing more information and variables to aid in future meta-analysis and also provide high-quality level 1 evidence studies.

## Conclusions

Our meta-analysis revealed that vitamin D deficiency is associated with an increased risk of surgery revision repair following RCT, emphasizing vitamin D's role in healing and tissue repair. No significant associations were found with post-operative pain or functional scores which could be related to the limited number of studies reporting such outcomes. Further high-quality studies are still needed to further understand the relation between vitamin D’s role to post-operative complications following RCR.
